# Liver development in Atlantic cod (*Gadus morhua* L.) larvae: Histomorphological analysis of biliary ABC transporters and hepatic vacuolization

**DOI:** 10.1007/s10695-025-01623-7

**Published:** 2025-12-22

**Authors:** Joachim Larsen Marthinsen, Kjell Inge Reitan, Elin Kjørsvik, Tora Bardal, Keshuai Li, Bruno Nunes, Amalie Munthe Vassbotn, Rolf Erik Olsen

**Affiliations:** 1 Department of Biology, Norwegian University of Science and Technology, 7491 Trondheim, Norway; 2 BioMar AS, Havnegata 9, 7010 Trondheim, Norway

**Keywords:** Atlantic cod larvae, *Gadus morhua*, Liver development, Abcb4, Abcb11, Hepatic vacuolization

## Abstract

Atlantic cod (*Gadus morhua*) larvae begin exogenous feeding while organ systems are still immature. Although the liver is morphologically differentiated at hatching, its functional development remains poorly understood. This study characterized histomorphological liver development in cod larvae using (1) monoclonal antibodies C219 and C494 to assess biliary secretory capacity across the hepatocyte canalicular membrane via ATP-binding cassette transporters, and (2) hepatic vacuolization as an indicator of energy storage maturation. Larvae were fed four formulated diets in a 2 × 2 factorial design from 17–61 days post-hatching (dph) to test effects of phospholipid level (~ 7 vs. 6 % dry matter) and bile salt supplementation (0 vs. 0.04 % dry matter). Only C219 labeled bile canaliculi, evident before first-feeding (2 dph) and peaking at 30 dph (9.2 mm standard length), suggesting enhanced capacity for phosphatidylcholine (Abcb4) and/or bile salt (Abcb11) efflux prior to metamorphic climax. Hepatic vacuolization remained low (2.3 %) until late metamorphosis, then increased 14-fold (35.9 %) in a sigmoid pattern with larval size. The steepest rise occurred at 17.7 mm standard length, marking the onset of hepatic lipid storage as larvae transition to juveniles. Dietary treatments did not significantly affect C219 labeling, vacuolization or histological biomarkers of nutritional status. These results indicate that cod liver functionality develops progressively during the larval stage and is largely unaffected by dietary phospholipid level and bile salt supplementation under the tested conditions.

**Supplementary Information:**

The online version contains supplementary material available at 10.1007/s10695-025-01623-7.

## Introduction

The Atlantic cod (*Gadus morhua*) is a commercially important marine teleost with altricial larvae that hatch at an early developmental stage, characterized by limited organ functionality and high nutritional sensitivity (Kjørsvik et al. 2004). Exogenous feeding must begin before the yolk is fully absorbed around 8–10 days post-hatching (dph) (Fossum 1986), as prolonged starvation beyond this point will cause irreversible damage to digestive tissues and ultimately mortality (Ellertsen et al. 1980; Kjørsvik et al. 1991). Developing digestive organs respond not only to food quantity but also to quality, which can affect the histomorphological organization of the liver, intestine and exocrine pancreas (Gisbert et al. 2008). In cod larvae, the liver was found to be particularly sensitive to dietary lipid composition, with hepatocyte cell size, nucleus size and mitochondrial membrane structure serving as reliable biomarkers of nutritional status (Wold et al. 2009).

The liver plays a central role in nutrient metabolism and homeostasis. In cod, the liver is morphologically differentiated at hatching, with hepatocyte cords arranged between sinusoids and bile canaliculi (Morrison 1993). These canaliculi drain bile into the gallbladder for storage and subsequent release into the intestinal tract, where bile salts (BSs) facilitate lipid digestion through emulsification and activation of bile salt-dependent lipase (BSDL) that hydrolyzes neutral lipids into absorbable units (Gjellesvik et al. 1992). The capacity to synthesize and secrete bile is therefore a key indicator of liver functionality in larval fish (Hoehne-Reitan and Kjørsvik 2004). However, little is known about the development of the biliary function in cod, and lipid digestibility has been suggested to be low during early developmental stages due to insufficient BS levels (Olsen et al. 1991).

Fish bile contains variable amounts of phosphatidylcholine (PC) (Moschetta et al. 2005), the major phospholipid (PL) coating the surface of lipoproteins that transport lipids to peripheral tissues (Tocher et al. 2008; Turchini et al. 2021). Deficiency in PC can impair lipid utilization and lead to accumulation of lipid droplets in enterocytes (Fontagné et al. 1998; Olsen et al. 2003). Since fish larvae have limited de novo PL synthesis (Teshima et al. 1987), an adequate dietary supply is critical for growth, survival and development of several species (Kanazawa et al. 1983; Geurden et al. 1995; Cahu et al. 2003; Seiliez et al. 2006; Hamza et al. 2008), including cod (Hansen et al. 2011). However, a tube-feeding experiment by Li et al. (2016) demonstrated that cod larvae can synthesize PC de novo from triacylglycerol (TAG) precursors at 30 dph and that the larval PC:TAG ratio increased when supplementing choline, a vitamin that must be obtained from the diet (Ren et al. 2025). Transcriptomic data further indicate a stable expression of PC synthesis genes in cod from hatching to 60 dph (Li et al. 2015). Together, these findings could suggest that the input of biliary PC from the immature cod larval liver is limited (Li et al. 2015, 2016), thereby increasing the reliance on dietary PC for efficient lipoprotein assembly in the intestine (Mansbach 1977).

Early studies proposed that biliary secretion in fish larvae occurs by exocytosis (Mani-Ponset et al. 1994; Diaz et al. 1997), but later findings indicate that this process is mediated by ATP-binding cassette (ABC) transporters (Zaja et al. 2008; Ellis et al. 2018). In mammals, transport of BS and PC across the hepatocyte canalicular membrane is mediated by ABCB11 and ABCB4, respectively (Morita and Terada 2014). Whereas ABCB11 appears to be highly conserved across vertebrates (Dean and Annilo 2005; Lukenbach et al. 2014), teleost Abcb4 may instead function primarily in xenobiotic transport (Zaja et al. 2008; Fischer et al. 2013). To date, however, these ABC transporters have not been studied in cod, and characterizing their localization and abundance during early ontogeny could provide important insight into liver maturation and biliary function.

A functional fish larval liver also synthesizes, stores and mobilizes nutrients to maintain nutritional homeostasis (Hoehne-Reitan and Kjørsvik 2004). In cod, hepatic energy reserves are initially low. Glycogen appears only after the yolk is resorbed (Kjørsvik et al. 1991), and lipid storage is minimal throughout the larval stage, suggesting that lipids are mainly used for energy and growth prior to metamorphosis (Wold et al. 2009). By contrast, hepatic lipids constitute the major energy reserve in cod juveniles and adults, enabling them to endure periods of low feeding activity (Black and Love 1986). Well-fed 6-month-old juveniles retain up to 60 % of assimilated lipids in the liver, mainly as TAG (93 %), which can comprise up to 70 % of the parenchymal tissue (Lie et al. 1986). However, the developmental onset of hepatic lipid accumulation during the larval-juvenile transition has not yet been described.

The main objective of this study was to characterize liver development in Atlantic cod larvae, with emphasis on (1) biliary secretory capacity, investigated by immunohistochemical analysis of Abcb4 and Abcb11, and (2) hepatic vacuolization as an indicator of maturation in energy storage (glycogen and/or lipids). To assess nutritional modulation of these processes, larvae were fed four formulated diets differing in PL level (high vs. low) and BS supplementation (present vs. absent) during co-feeding with live feeds (17–34 dph) and up to 60 dph. Nutritional status was further evaluated using histological biomarkers including hepatocyte nucleus and cell size, enterocyte height, and mucosal fold height. Because Abcb4 and Abcb11 have not previously been studied in cod, we additionally applied immunohistochemistry to whole-larval sections to provide the first description of their tissue distribution in this species.

## Materials and methods

### Experimental design

Cod eggs (Tromsø Aquaculture Research Station AS, Tromsø, Norway) were incubated in 12 × 200 L larval rearing tanks at an initial density of ~ 125 eggs L^−1^. After hatching, larvae were reared in triplicate groups and fed rotifers (*Brachionus plicatilis*) from 3–19 dph, *Artemia sp*. from 15–34 dph, and one of four formulated diets (BioMar AS, Nersac, France) from 17–60 dph. The experiment followed a 2 × 2 factorial design, with diets differing in PL level and the inclusion of taurocholate as a BS supplement (Table 1). The amounts of fish oil, marine PL and vegetable PL added to the diets were adjusted while keeping a constant total lipid content (22 % dry matter, dm). Further details on larval rearing conditions and nutritional composition of the experimental diets are provided in Marthinsen et al. (2025).

**Table 1:** Experimental diets HPL, HPL-BS, LPL and LPL-BS defined by phospholipid level and bile salt supplementation

	HPL	HPL-BS	LPL	LPL-BS
*Factor*				
PL level	High	High	Low	Low
BS supplementation	No	Yes	No	Yes
*Ingredients (% dm)*				
Fish meal	89.00	88.96	89.50	89.46
Fish oil	3.00	3.00	6.00	6.00
Marine PL	2.00	2.00	-	-
Vegetable PL	6.00	6.00	4.50	4.50
Taurocholate^1^	-	0.04	-	0.04
*Phospholipids (% dm)*^2^				
PC	3.20	3.28	2.75	2.86
1-LPC	0.09	0.09	0.10	0.07
2-LPC	0.50	0.54	0.43	0.42
PI	0.82	0.96	0.71	0.81
PA	0.42	0.44	0.35	0.36
LPA	0.04	0.05	0.02	0.04
Other^3^	1.84	2.01	1.35	1.55
** Sum PL**	**6.91**	**7.37**	**5.71**	**6.10**
*Bile salt (% dm)*				
Taurocholate^4^	-	0.05	-	0.02

*LPA* lysophosphatic acid, *LPC* lysophosphatidylcholine, *PA* phosphatic acid, *PI* phosphatidylinositol

^1^Bile salt added as ≥ 95% taurocholic acid sodium salt hydrate (T4009, Merck, Germany)

^2^Phospholipids analyzed by quantitative ^31^P-NMR spectroscopy (Spectral Service AG, Cologne, Germany)

^3^Other phospholipids may include phosphatidylethanolamine, lysophosphatidylethanolamine

N-acyl-phosphatidylethanolamine, phosphatidylserine and sphingomyelin

^4^Taurocholate estimated by ^1^H-NMR spectroscopy as the difference in total sterols between diets with bile salt (HPL-BS and LPL-BS) and without bile salt (HPL and LPL) (Spectral Service AG, Cologne, Germany)

### Sampling

Larvae were sampled regularly throughout the rearing period (2, 8, 15, 30, 45, 52, 60 and 61 dph) for immunohistochemical and histological analyses. At each sampling point, approximately ten larvae were randomly collected from each tank using a wide-bore transparent tube to minimize handling stress and capture larvae at all depths of the tank. At 60 and 61 dph, larvae were instead sampled using a hand net due to their larger size and being more difficult to catch. Sampled larvae were euthanized with an anesthetic overdose (200–400 mg L^−1^) of tricaine methanesulfonate (MS222, ScanVacc AS, Norway) and fixed in 4 % paraformaldehyde in phosphate buffered saline (PBS) (w/v). Immunohistochemistry samples were left at room temperature overnight before being transferred to 70 % ethanol, and all samples were stored at 4 °C until further processing. Measurements of standard length (SL) of fixed larvae were performed using ImageJ (Schneider et al. 2012) on photographs (Zen Core; AxioCam ERc 5 s, Carl Zeiss AG, Germany) captured under a stereomicroscope (Leica MZ12.5, Leica Microsystems, Germany). Dead larvae were removed and counted once or twice per day between 20 and 60 dph to estimate survival.

### Immunohistochemistry

The monoclonal antibody C219 (ALX-801–002, Enzo Life Sciences, USA) binds to highly conserved amino acid sequences shared by mammalian ABCB1 and ABCB4 (Georges et al. 1990), while also recognizing mammalian ABCB11 (Childs et al. 1995). ABCB1 is expressed in several cell types, including hepatocytes, where it localizes to the canalicular membrane and functions as a multidrug export pump (Zhou 2008). To distinguish C219 labeling of Abcb1 from that of Abcb4/Abcb11, the monoclonal antibody C494 (ALX-801–003-C100, Enzo Life Sciences, USA) was used in addition to C219. C494 is specific for ABCB1 (Georges et al. 1990), while also cross-reacting with pyruvate carboxylase (Rao et al. 1994).

Fixed samples were dehydrated (Leica TP1020, Leica Biosystems, Germany), embedded in paraffin (Tissue-Tek® III Embedding Wax, Sakura, UK), and sectioned at 4 µm thickness (Leica HistoCore AUTOCUT, Leica Biosystems, Germany). Sections were mounted on silane-coated slides, dried at 37 °C overnight, deparaffinized, and rehydrated. Heat induced epitope retrieval was performed by antigen demasking in a pressure cooker with Tris–EDTA (10 mM/1 mM, pH 9.0) for 5 min. Sections were incubated overnight at 4 °C with C219 or C494, each diluted 1:500 (v/v) with normal horse serum (S091H, Biowest) and PBS (1:9, v/v). C219 labeling was visualized using alkaline phosphatase (ImmPRESS®-AP Horse Anti-Mouse IgG Polymer Detection Kit) and Vector Red as the chromogen (ImmPACT® Vector® Red Substrate Kit). Horseradish peroxidase and 3,3’-diaminobenzidine (DAB) (ImmPRESS® HRP Horse Anti-Mouse IgG PLUS Polymer Kit) were used for C494. All reagents were obtained from Vector Laboratories, Inc., USA. One negative control section without primary antibody was included on each slide. Sections were mounted, digitalized (NanoZoomer SQ, Hamamatsu Photonics, Japan), and accessed in NDP.view2 (Hamamatsu Photonics, Japan).

Labeling of bile canaliculi was quantified using the pixel classifier in QuPath (Bankhead et al. 2017). Quantification was performed on three transverse liver sections per larva, avoiding vascular tissue as much as possible. Mean values were normalized according to Eq. (1) and then compared across age groups (*n* = 6 larvae at 8, 15, 30, 45 and 60 dph) and diet groups at 60 dph (*n* = 2 larvae per tank):

1 $$\mathrm{L}_n = \frac{\mathrm{L}_m}{\left(1 - \frac{\overline{\mathrm{V}}}{100\,\%}\right)}$$

where L_*n*_ and L_*m*_ represent normalized and measured labeling percentages (% area), respectively, and V̅ is the mean hepatic vacuolization (% area) per age and diet group. Normalization was applied to account for hepatocyte hypertrophy caused by glycogen and lipid accumulation (Caballero et al. 1999; Fishelson and Becker 2001), which reduces the relative area available for canalicular labeling per area unit of liver tissue.

### Histology

Histological analysis was conducted at 15, 30, 45, 52 and 61 dph (*n* = 2 larvae per tank and age group). Fixed samples were washed 3 × 5 min in PBS, dehydrated in ethanol (50 %, 70 % and 2 × 96 %, 10 min each), and embedded in glycol methacrylate (Technovit® 7100, Kulzer, Germany). Larvae were sectioned longitudinally (15–45 dph) or transversely (52 and 60 dph) at 2 µm thickness (Leica HistoCore AUTOCUT, Leica Biosystems, Germany). Sections were stained with 0.05 % Toluidine Blue (89640, Sigma-Aldrich, USA) in 1 % sodium tetraborate buffer (w/v) for 15 s. Periodic acid-Schiff (PAS) staining was performed using standard protocols (Electron Microscopy Sciences, Hatfield, USA), except using Mayer’s hematoxylin as counterstain. PAS-positive staining of glycogen was confirmed by enzymatic digestion with 1 % amylase (A3176, Sigma-Aldrich, USA) at 37 °C for 6 h. Sections were mounted and digitalized on a NanoZoomer SQ (Hamamatsu Photonics, Japan). Morphometric measurements were performed exclusively on Toluidine Blue-stained sections.

Liver morphology was assessed in QuPath (Bankhead et al. 2017) using one or more random areas (250 × 250 µm^2^) encompassing at least 200 hepatocyte nuclei (Wold et al. 2009). Hepatocyte nucleus size was measured from all nuclei containing visible nucleoli. Hepatocyte cell size was calculated as total liver area divided by the number of nuclei. Area fractions of hepatic vacuolization and vascularization were estimated in ImageJ using a 16 × 16 point grid (Gundersen et al. 1988; Schneider et al. 2012). The area fraction of hepatic vascularization was subtracted from the total liver area before calculating hepatocyte cell size and hepatic vacuolization.

For morphological evaluation of the intestine, enterocyte height and mucosal fold height were measured at ten and five randomly selected locations per larva, respectively. The measurements were performed with NDP.view2 (Hamamatsu Photonics, Japan), without differentiating between intestinal regions.

### Statistics

Statistical analyses were performed in R (v4.3.2; R Core Team 2023) using a significance level of α = 0.05. Assumptions of normality and homogeneity of variance were assessed using Shapiro–Wilk’s and Levene’s tests, respectively. Data are reported as mean ± standard error of mean (SEM) and visualized with ‘ggplot2’ (v3.5.1; Wickham 2016) and ‘ggpubr’ (v0.6.0; Kassambara 2023).

Diet effects on standard length, bile canalicular labeling and all histological parameters, except hepatic vacuolization, were analyzed using linear mixed-effects models (‘lme4’ v1.1.37; Bates et al. 2015) with PL, BS and their interaction (PL × BS) as fixed factors and tank as a random effect, allowing individual larvae to serve as replicates while accounting for tank-level variation. Significance of fixed factors was evaluated using two-way ANOVA, while post hoc comparisons of estimated marginal means (EMMs) were conducted using the *emmeans* function with Tukey adjustment for multiple testing (‘emmeans’ v1.11.2; Lenth 2025). Differences in survival were analyzed using normal linear models with two-way ANOVA and Tukey’s HSD post hoc test.

Hepatic vacuolization was modelled using beta regression with a logit link (Cribari-Neto and Zeileis 2010). Diet effects were tested using generalized linear mixed-effects models (‘glmmTMB’ v1.1.11; Brooks et al. 2017) with the same model structure as described above. Significance of fixed factors was assessed using likelihood ratio tests comparing nested models, and post hoc comparisons were performed using *emmeans*. The relationship between hepatic vacuolization (V) and larval size (SL) was assessed using nonlinear least squares regression with a four-parameter Gompertz (1825) growth function (Gibson et al. 1987; Tjørve and Tjørve 2017) (Eq. 2):

2 $$\mathrm{V} = B + A\,\mathrm{exp}\!\left(-\mathrm{exp}\!\left(-k(\mathrm{SL} - \mathit{IP})\right)\right)$$

where *B* is the lower asymptote (minimum vacuolization), *A* the growth magnitude (maximum increase in vacuolization), *k* the growth rate constant (maximum rate of increase in vacuolization) and *IP* the inflection point (SL when the slope is equal to *k*). Model parameters were compared between diet groups using pairwise Wald tests, with *p*-values corrected for multiple testing according to Benjamini and Hochberg (1995). Model fit was evaluated with Nagelkerke’s (1991) coefficient of determination (*r*^2^_NK_).

Differences within diet groups were analyzed using one-way ANOVA with larval age (dph) as a factor, followed by Tukey’s HSD post hoc test. For hepatic vacuolization, age groups were compared using generalized linear models with beta distribution and logit link, with post hoc comparisons obtained via *emmeans*. Larvae were treated as individual replicates for all within-group comparisons.

## Results

### Growth and survival

Larval SL increased from an average of 5.9 ± 0.1 mm at 15 dph to 23.4 ± 0.5 mm at the end of the trial. No size differences were observed between diet groups (Table 2), but larvae fed the LPL-BS diet tended to be shorter than those fed HPL-BS at 30 dph (EMMs Tukey post hoc test, *p* = 0.071). Survival from 20 to 60 dph was unaffected by the dietary treatments.

**Table 2:** Standard length and survival of *G. morhua* larvae fed the diets HPL, HPL-BS, LPL and LPL-BS from 17–60 dph

Parameter	Dph	HPL	HPL-BS	LPL	LPL-BS	*p-*values		
						PL	BS	PL × BS
Standard length (mm)	15	6.0 ± 0	6.0 ± 0.2	5.7 ± 0.2	6.0 ± 0.1	-	-	-
	30	9.3 ± 0.2	9.8 ± 0.2	9.5 ± 0.2	8.9 ± 0.1	0.18	0.69	0.028
	45	13.5 ± 0.5	12.7 ± 0.3	13.3 ± 0.5	12.9 ± 0.4	0.99	0.21	0.63
	52	18.6 ± 1.2	18.6 ± 0.5	16.9 ± 0.5	17.5 ± 1.0	0.12	0.74	0.83
	60/61	22.9 ± 1.3	23.3 ± 0.9	24.3 ± 0.9	23.1 ± 0.9	0.49	0.75	0.36
Survival (%)	20	100	100	100	100	-	-	-
	30	90 ± 1	90 ± 2	88 ± 2	88 ± 3	0.37	0.34	0.24
	45	80 ± 0	72 ± 5	75 ± 4	76 ± 3	0.81	0.55	0.14
	52	74 ± 2	68 ± 4	71 ± 4	72 ± 4	0.89	0.83	0.22
	60	53 ± 4	60 ± 2	42 ± 10	50 ± 4	0.14	0.59	0.66

Values are given as mean ± SEM (*n* = 6 and 12 for standard length at 15–52 dph and 60/61 dph, respectively; *n* = 3 for survival). Within a row, *p*-values indicate the effect of PL level, BS supplement and their interaction (PL × BS) (two-way ANOVA)

### Immunohistochemistry

#### Monoclonal antibody C219

**Fig. 1:**
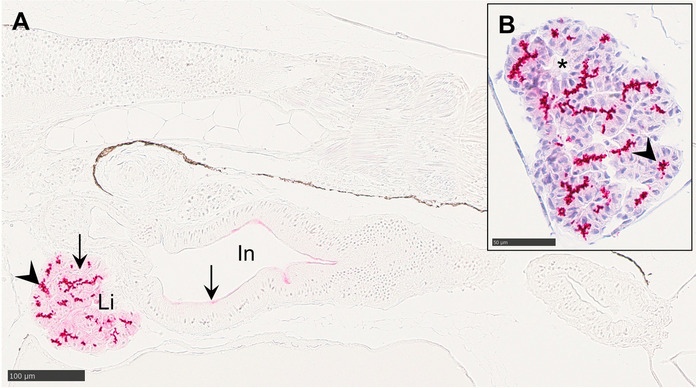
Longitudinal paraffin-embedded tissue sections of *G. morhua* at 2 dph incubated with monoclonal antibody C219. Positive labeling is visualized with alkaline phosphatase and Vector Red. (**A**) High intensity of granulated and clustered labeling in the liver (arrowhead), and weaker labeling in hepatocyte cytoplasm and along the apical membrane of enterocytes in the intestine (arrows). (**B**) Labeling clusters in the liver consist of adjacent hepatocytes with polarized membrane labeling oriented towards bile canaliculi (arrowhead) and are absent near vascular structures (*). The section is counterstained with Mayer’s hematoxylin to provide structural information. In, intestine; Li, liver. Scale bar sizes are indicated on each image

**Fig. 2:**
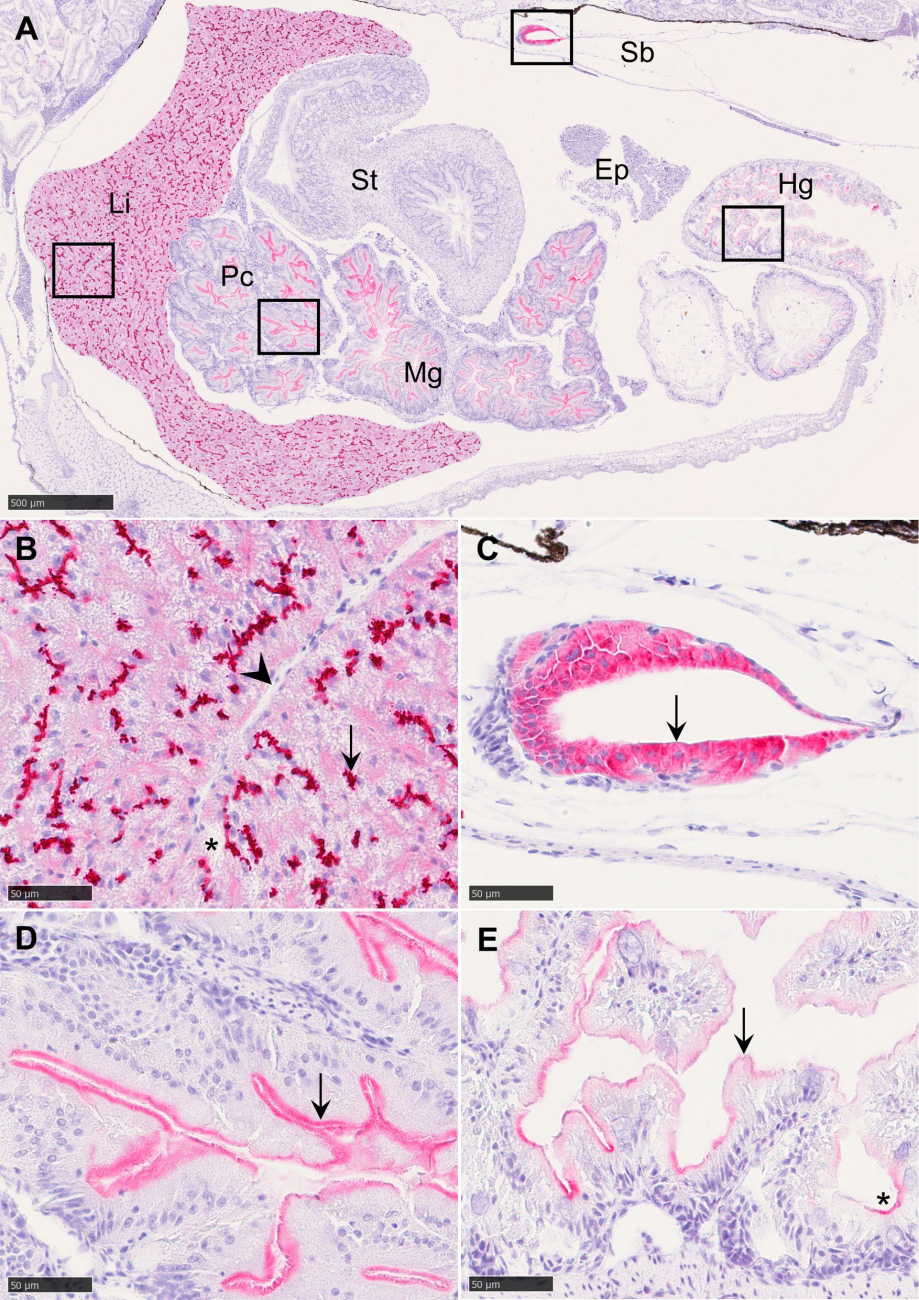
Longitudinal paraffin-embedded tissue section of *G. morhua* at 60 dph incubated with monoclonal antibody C219 and counterstained with Mayer’s hematoxylin. Positive labeling (arrows) is visualized with alkaline phosphatase and Vector Red. (**A**) Overview of tissues displaying positive labeling. The marked squares are magnified in images B-E. (**B**) High intensity of granulated and clustered labeling in the liver shows in bile canaliculi and is absent near vascular structures (arrowhead). Numerous small vacuoles (*) are present in hepatocytes. (**C**) Labeling in the gas gland of the swim bladder. (**D**) Labeling in the apical membrane of enterocytes in *pyloric caeca*. (**E**) Labeling in the apical membrane of enterocytes in the hindgut. The crypts (*) of mucosal folds show higher labeling intensity than their tips. Ep, exocrine pancreas; Hg, hindgut; Li, liver; Mg, midgut; Pc, *pyloric caeca*; Sb, swim bladder; St, stomach. Scale bar sizes are indicated on each image

Incubation of whole-larval sections with C219 revealed positive labeling in the liver and on the apical membrane of enterocytes at 2 dph, although labeling in the intestine was patchy and showed no consistent pattern along its length (Fig. 1A). In the liver, two distinct labeling patterns were identified across developmental stages (2–60 dph) (Fig. 2A): (1) a diffuse, low-intensity signal in the hepatocyte cytoplasm and (2) an intense, polarized granular labeling at hepatocyte membranes bordering structures identified as bile canaliculi (Fig. 1B, Fig. 2B). The granular labeling pattern in the liver was consistently absent near vascular structures. Labeling was also detected in the gas gland of the swim bladder (Fig. 2C). By 60 dph, when the digestive system was completely morphologically differentiated, the intestinal signal was stronger in the midgut and *pyloric caeca* (Fig. 2D) compared to the hindgut, where labeling appeared more pronounced in the crypts of the mucosal folds than at their tips (Fig. 2E). Negative control sections confirmed that labeling was specific and not due to endogenous alkaline phosphatase activity (Fig. S1).

**Fig. 3:**
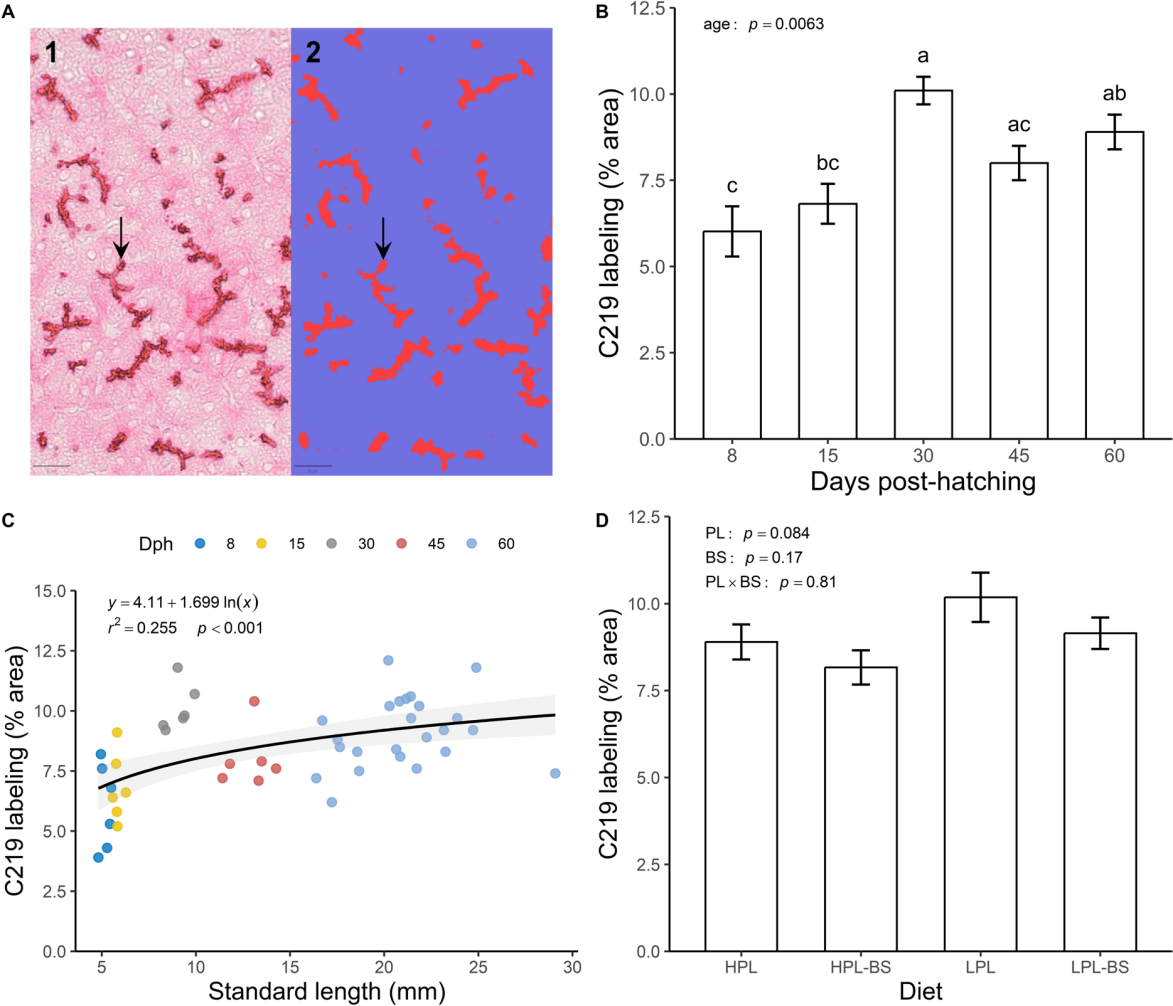
C219 labeling of bile canaliculi in *G. morhua* liver (% area). (**A**) Detection of positive labeling (arrows) using the pixel classifier in QuPath. Image (1) shows a liver section under the microscope, and image (2) highlights classified positive areas. Scale bars equal to 20 µm. (**B**) Labeling at different days post-hatching. The *p*-value indicates the effect of larval age (one-way ANOVA). Different letters above the bars denote significant differences among the age groups (Tukey’s HSD post hoc test, *p* < 0.05). (**C**) Logarithmic regression between labeling and larval standard length (*n* = 48). The *p*-value indicates the significance of the model. (**D**) Labeling at 60 dph in larvae fed the diets HPL, HPL-BS, LPL and LPL-BS from 17–60 dph. The *p*-values indicate the effects of PL level, BS supplement and their interaction (PL × BS) (two-way ANOVA). Values in B and D are given as means ± SEM (*n* = 6)

Positive C219 labeling of bile canaliculi in the liver was accurately identified by the pixel classification model (Fig. 3A). Labeling ranged from 3.9 to 12.1 % of the total liver area and was significantly affected by age, increasing at 30 dph compared to both 8 and 15 dph (Tukey’s HSD post hoc test, *p* ≤ 0.0025) (Fig. 3B). No further increase in labeling was observed beyond 30 dph, but levels remained significantly higher at 60 dph than at 8 dph (*p* = 0.0087). Labeling showed a weak logarithmic relationship with larval size, with a steep increase between 5–10 mm SL and little change at larger sizes (Fig. 3C) Diet had no significant effect on labeling at 60 dph (Fig. 3D), although larvae fed low-PL diets showed a tendency toward higher labeling (9.7 ± 0.4 %) than those fed high-PL diets (8.5 ± 0.4 %) (EMMs Tukey post hoc test, *p* = 0.082). Normalization details are provided as supplementary information (Table S1).

#### Monoclonal antibody C494

**Fig. 4:**
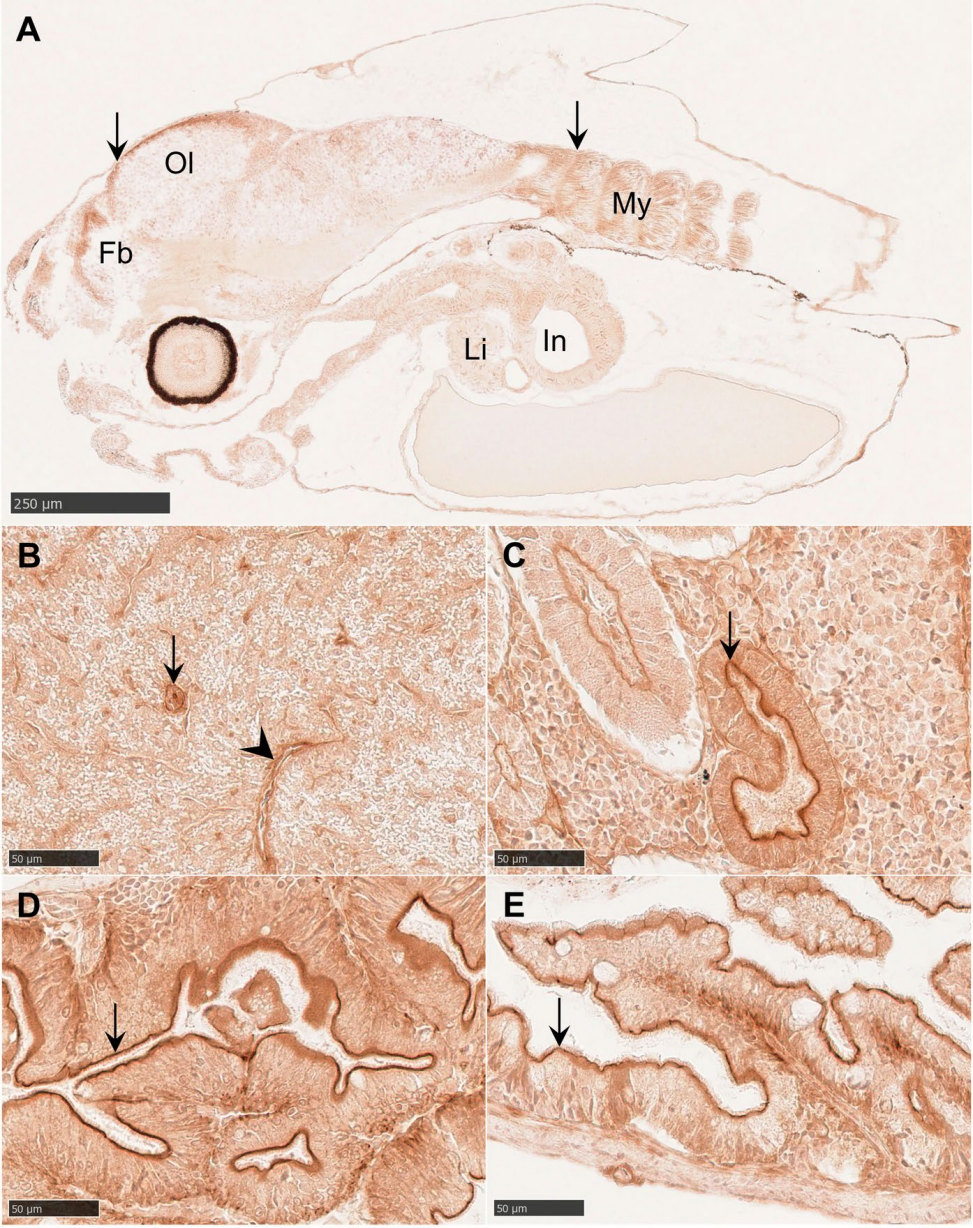
Longitudinal tissue sections of *G. morhua* incubated with monoclonal antibody C494. Positive labeling is visualized with horseradish peroxidase and DAB. (**A**) Larva at 2 dph displaying weak labeling in all tissues and higher labeling intensity anterodorsally in the forebrain and optic lobe, and in myotome muscle fibers (arrows). (**B**) Labeling in bile ductule epithelium (arrow) and endothelial cells of vascular tissue (arrowhead) in the liver at 60 dph. (**C**) Labeling in the apical membrane of epithelium in a kidney tubule (arrow) at 60 dph. (**D**) Labeling in the apical membrane of enterocytes (arrow) in *pyloric caeca* at 60 dph. (**E**) Labeling in the apical membrane of enterocytes (arrow) in the hindgut at 60 dph. Fb, forebrain; Li, liver; In, intestine; My, myotome; Ol, optic lobe. Scale bar sizes are indicated on each image

Whole-larval sections incubated with C494 showed a weak but ubiquitous labeling pattern. In pre-feeding larvae (2 dph), higher intensity was observed anterodorsally in the forebrain and optic lobe, as well as in myotome muscle fibers (Fig. 4A). At this stage, the liver displayed only a faint cytoplasmic signal in hepatocytes, whereas older larvae also exhibited slightly stronger labeling in bile ductule epithelium and vascular endothelium (Fig. 4B). No distinct labeling of bile canaliculi was detected at any stage. The overall strongest labeling intensity was identified along the apical membranes of epithelial cells in kidney tubules and in enterocytes at 60 dph. Whereas labeling varied among individual kidney tubules (Fig. 4C), it was consistent in the intestine with no differences between the midgut and the hindgut (Fig. 4D, E). Negative controls showed no positive labeling (Fig. S2).

### Histology

#### Liver

**Fig. 5:**
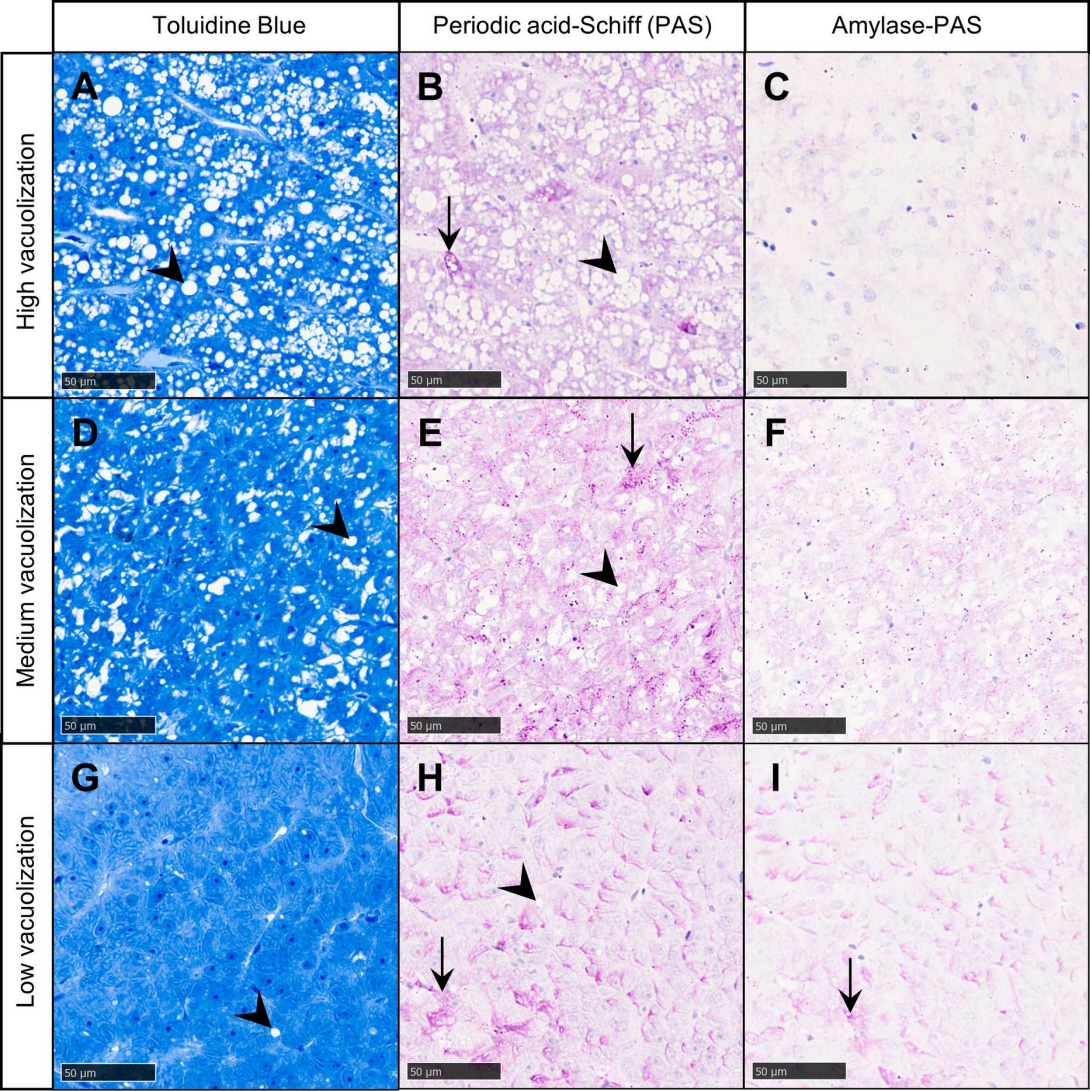
Hepatic vacuolization and energy storage profiles in liver of *G. morhua* at 52 dph. Representative livers displaying high (top row), medium (middle row), and low (bottom row) vacuolization. Sections are embedded in Technovit, cut transversally and stained with Toluidine Blue to visualize lipid droplets (arrowheads) (left column: **A**, **D**, **G**), Periodic acid-Schiff (PAS) to detect glycogen and other carbohydrate-rich substances (arrows) (middle column: **B**, **E**, **H**), and amylase-PAS to confirm glycogen specificity by enzymatic digestion (right column: **C**, **F**, **I**). Sections stained with PAS and amylase-PAS are counterstained with Mayer’s hematoxylin. Scale bars equal to 50 µm

Liver histology parameters did not differ significantly among diet groups at any point during the experiment. A significant effect of BS supplementation was detected for hepatic vascularization at 61 dph, but pairwise comparisons were not significant (EMMs Tukey post hoc test, *p* ≥ 0.26) (Table 3). Within-group comparisons showed shrinking hepatocyte nuclei between 15 and 30 dph, though this was not significant in the LPL group (Tukey’s HSD post hoc test, *p* = 0.091). Nucleus size recovered or exceeded initial values by 52 dph before declining again toward the end of the trial. This late decline was significant only for LPL-BS larvae, whose nuclei at 61 dph were smaller than at both 52 and 15 dph (Tukey’s HSD post hoc test, *p* ≤ 0.023). Hepatocyte cell size followed a similar early decline but then doubled between 45 and 61 dph. This hypertrophy coincided with extensive hepatic vacuolization, primarily reflecting an accumulation of lipid droplets (Fig. 5A, D, G). Livers with high vacuolization exhibited weaker PAS staining (Fig. 5B) than those with medium (Fig. 5E) or low vacuolization (Fig. 5H), and PAS reactivity was markedly reduced after amylase digestion (Fig. 5C, F, I), indicating that glycogen was the main PAS-positive compound. Irrespective of BS supplementation, vacuolization tended to be higher in larvae fed high-PL diets (22.0 ± 4.2 %) than those fed low-PL diets (12.0 ± 2.8 %) at 52 dph, but this difference was not statistically significant (EMMs Tukey post hoc test, *p* = 0.068).

**Table 3:** Liver histology parameters in *G. morhua* larvae fed the diets HPL, HPL-BS, LPL and LPL-BS from 17–60 dph

Parameter	Dph	HPL	HPL-BS	LPL	LPL-BS	*p*-values		
						PL	BS	PL × BS
Hepatocyte nucleus size (µm^2^)	15	19.7 ± 0.5^b^	22.5 ± 0.8^a^	17.7 ± 0.3^bc^	21.5 ± 0.8^a^	-	-	-
	30	16.1 ± 0.4^c^	16.0 ± 0.5^b^	15.6 ± 0.4^c^	15.9 ± 0.5^b^	0.57	0.83	0.71
	45	18.1 ± 0.8^bc^	18.9 ± 0.8^ab^	17.2 ± 0.8^c^	17.6 ± 0.3^b^	0.13	0.40	0.80
	52	23.5 ± 1.3^a^	22.6 ± 1.0^a^	21.5 ± 0.2^a^	22.5 ± 0.8^a^	0.27	0.95	0.32
	61	20.3 ± 0.9^ab^	20.8 ± 1.3^a^	19.9 ± 0.9^ab^	18.1 ± 1.0^b^	0.14	0.55	0.29
Hepatocyte cell size (µm^2^)	15	356 ± 1^c^	385 ± 25^bc^	310 ± 16^ cd^	377 ± 16^b^	-	-	-
	30	274 ± 11^d^	311 ± 20^c^	264 ± 17^d^	276 ± 9^c^	0.27	0.25	0.55
	45	402 ± 33^c^	402 ± 31^bc^	350 ± 15^c^	386 ± 26^b^	0.26	0.52	0.52
	52	575 ± 41^b^	486 ± 32^b^	458 ± 28^b^	501 ± 26^b^	0.13	0.49	0.055
	61	790 ± 49^a^	853 ± 94^a^	813 ± 54^a^	829 ± 103^a^	0.99	0.65	0.79
Hepatic vacuolization (% area)	15	0.8 ± 0.1^c^	1.3 ± 0.2^b^	0.6 ± 0.1^c^	0.6 ± 0.2^c^	-	-	-
	30	1.8 ± 0.4^c^	1.7 ± 0.8^b^	1.5 ± 0.4^bc^	1.6 ± 0.4^c^	1.0	1.0	0.28
	45	2.1 ± 0.5^c^	8.1 ± 3.3^b^	2.9 ± 0.7^bc^	3.5 ± 1.4^c^	1.0	0.51	0.18
	52	22.3 ± 7.2^b^	21.6 ± 5.1^a^	8.2 ± 4.3^b^	15.7 ± 3.0^b^	0.11	0.24	0.16
	61	34.6 ± 1.1^a^	33.3 ± 2.6^a^	37.2 ± 1.8^a^	35.5 ± 1.1^a^	1.0	1.0	1.0
Hepatic vascularization (% area)	15	5.7 ± 0.5^bc^	5.3 ± 0.7^b^	6.5 ± 0.9^b^	6.1 ± 0.5^b^	-	-	-
	30	11.8 ± 0.7^a^	11.9 ± 0.7^a^	14.0 ± 1.3^a^	13.4 ± 1.5^a^	0.18	0.86	0.80
	45	11.8 ± 1.1^a^	11.4 ± 1.9^a^	14.3 ± 1.4^a^	14.9 ± 1.2^a^	0.050	0.94	0.72
	52	4.8 ± 0.5^ cd^	6.1 ± 1.5^b^	6.0 ± 0.7^b^	5.6 ± 1.0^b^	0.78	0.77	0.53
	61	8.7 ± 0.9^ab^	7.1 ± 0.6^ab^	8.9 ± 1.0^b^	6.7 ± 0.6^b^	0.88	0.024	0.69

Values are given as mean ± SEM (*n* = 6). Within a row, *p*-values indicate the effect of PL level, BS supplement and their interaction (PL × BS) (two-way ANOVA for hepatocyte nucleus size, hepatocyte cell size and hepatic vascularization; likelihood ratio tests for hepatic vacuolization). Within a column, different superscript letters indicate significant differences between age groups for a given parameter (Tukey’s HSD or EMMs Tukey post hoc test, *p* < 0.05)

**Fig. 6:**
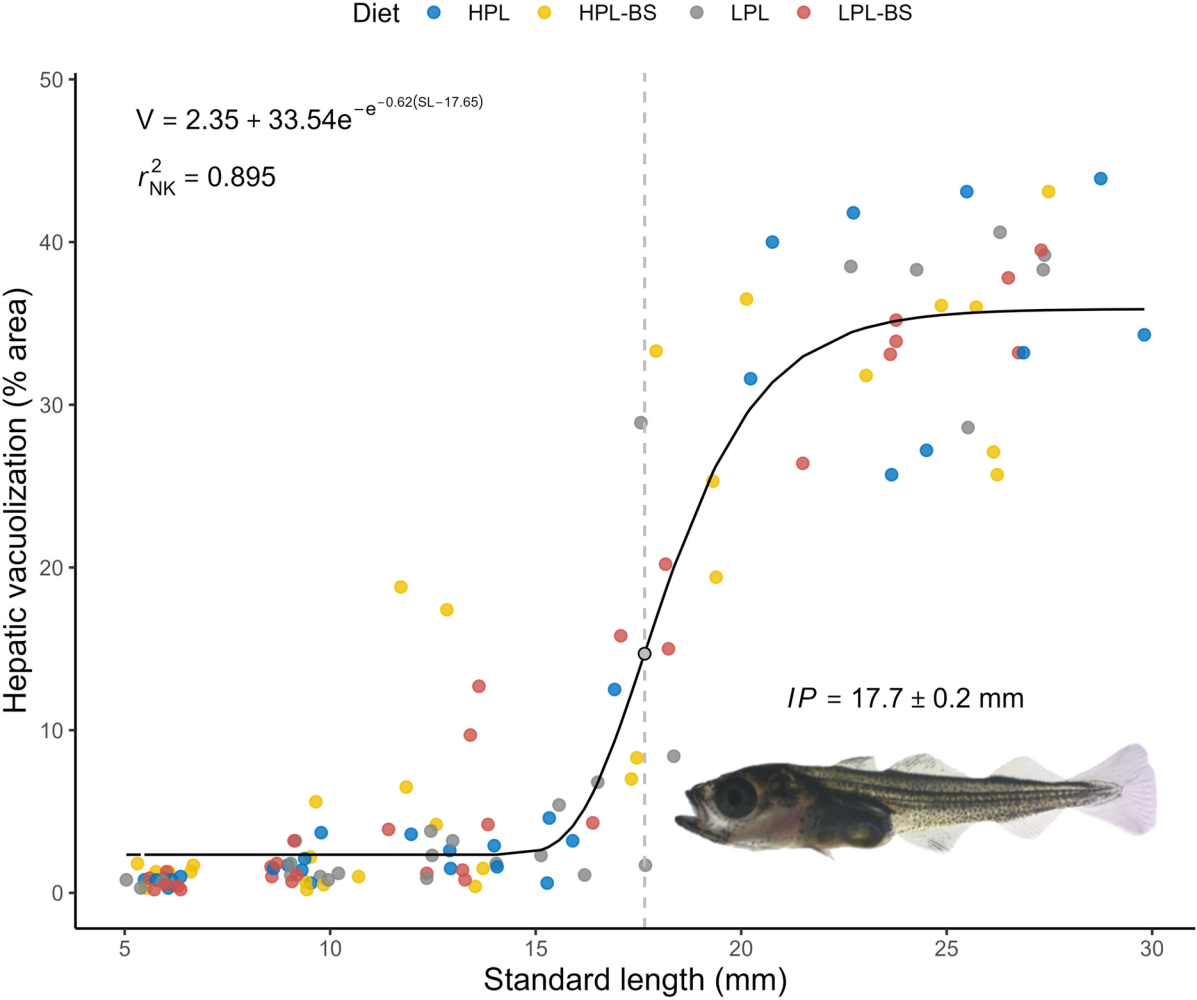
Relationship between hepatic vacuolization (V) and standard length (SL) in *G. morhua* from 15–61 dph across all diet groups, modelled with a Gompertz growth function (*n* = 120). The vertical dashed line marks the inflection point (*IP*), where the rate of increase in V is maximal. The superimposed image illustrates representative larval morphology at the *IP*

Hepatic vacuolization exhibited a sigmoid relationship with larval SL, as revealed by nonlinear least squares regression using a four-parameter Gompertz growth function (Fig. 6). Vacuolization remained low in small larvae (*B* = 2.35 ± 0.57 % area) and increased sharply above ~ 15 mm SL, reaching a 14-fold maximum in the largest individuals (*A* = 33.54 ± 1.27 % area). The rate of increase in vacuolization peaked at 17.7 ± 0.2 mm SL (*k* = 0.62 ± 0.12 % area mm^−1^) and plateaued beyond ~ 23 mm SL. Diet had a negligible effect on model fit (∆*r*^2^_NK_ = 0.015), with no significant differences in parameter estimates between the groups (Table 4).

**Table 4:** Estimated parameters from Gompertz growth functions describing the relationship between hepatic vacuolization and standard length in *G. morhua* fed the diets HPL, HPL-BS, LPL and LPL-BS from 17–60 dph (*r*^2^_NK_ = 0.910). The lower asymptote (*B*) is fixed across diet groups as it is based on measurements taken prior to the experiment (15 dph)

Parameter	HPL	HPL-BS	LPL	LPL-BS
Lower asymptote, *B* (% area)	2.18 ± 0.57	2.18 ± 0.57	2.18 ± 0.57	2.18 ± 0.57
Maximum growth magnitude, *A* (% area)	33.68 ± 1.83	29.32 ± 1.64	36.76 ± 3.41	41.10 ± 7.67
Growth rate constant, *k* (% area mm^−1^)	1.06 ± 0.88	7.61 ± 7.79	0.44 ± 0.19	0.22 ± 0.08
Inflection point, *IP* (mm)	17.08 ± 0.40	17.50 ± 0.08	18.35 ± 0.62	18.23 ± 1.28

Values are given as mean ± SEM (*n* = 30)

#### Intestine

**Fig. 7:**
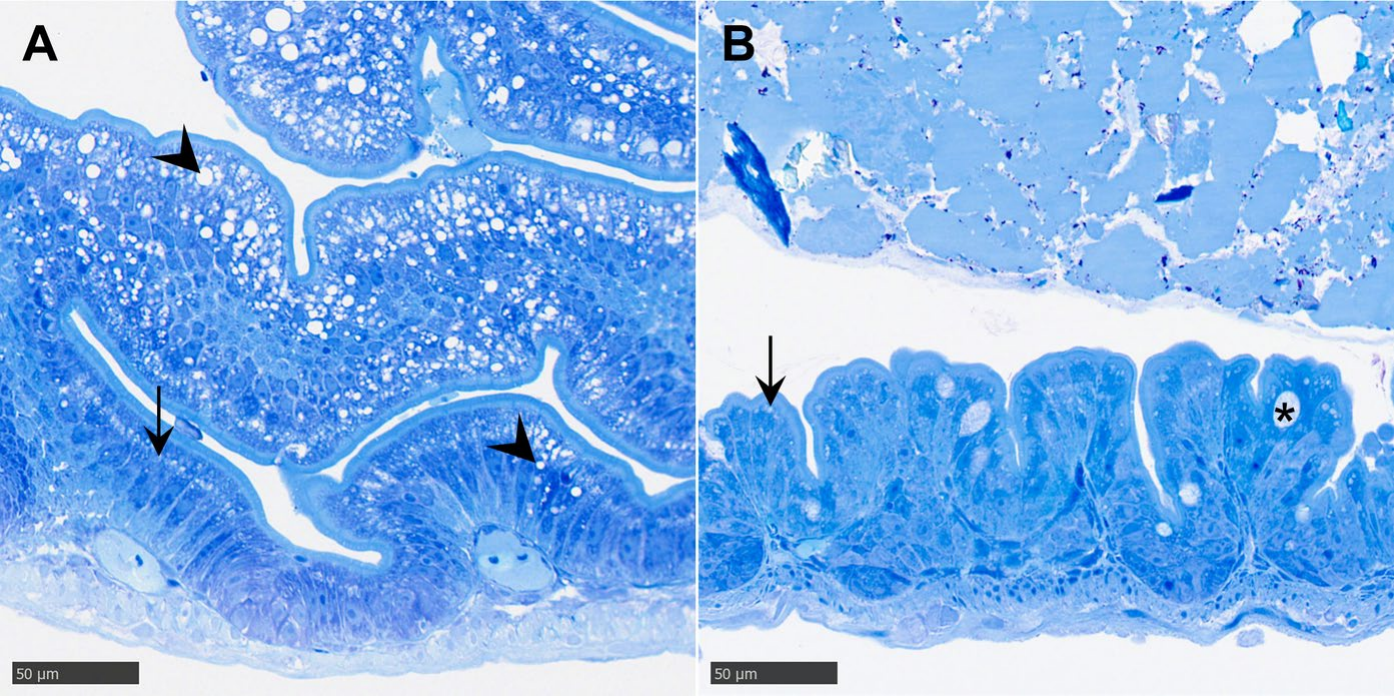
Transverse sections of intestine in *G. morhua* at 52 dph. (**A**) Midgut enterocytes showing high accumulation of large lipid droplets (1–10 µm) (arrowheads) and numerous small supranuclear vesicles (arrow). (**B**) Midgut enterocytes with no visible lipid droplets and few supranuclear vesicles. Note goblet cells (*) in the intestinal mucosa. Sections are embedded in Technovit and stained with Toluidine Blue. Scale bars equal to 50 µm

Dietary treatments had no significant effects on enterocyte height or mucosal fold height (Table 5). Enterocyte height increased by ~ 36 % on average across diet groups from 15 to 30 dph and remained stable thereafter, except in the HPL-BS group, which also showed an increase from 30 to 61 dph (Tukey’s HSD post hoc test, *p* = 0.026). Mucosal fold height increased two- to threefold between 15 and 52 dph, with no additional growth observed at 61 dph. Large intracellular lipid droplets (1–10 µm) appeared from 45 dph onwards, mainly at the apical end of midgut enterocytes and confined to discrete areas within the mucosal layer (Fig. 7A). Lipid droplets were present in 36 % of larvae sampled between 45 and 61 dph, while most larvae in this period showed no signs of accumulation (Fig. 7B). Their occurrence was unrelated to diet and showed no association with hepatic vacuolization. Among larvae with SL ≥ 17.7 mm (Fig. 6), hepatic vacuolization levels were similar between individuals with (32.8 ± 2.0 %) (*n* = 12) and without (31.2 ± 2.1 %) (*n* = 24) visible intestinal lipid droplets.

**Table 5:** Intestinal histology parameters in *G. morhua* larvae fed the diets HPL, HPL-BS, LPL and LPL-BS from 17–60 dph

Parameter	Dph	HPL	HPL-BS	LPL	LPL-BS	*p*-values		
						PL	BS	PL × BS
Enterocyte height (µm)	15	25.9 ± 0.8^b^	28.6 ± 1.2^c^	23.4 ± 1.8^b^	26.7 ± 1.7^b^	-	-	-
	30	36.0 ± 0.9^a^	35.6 ± 1.0^b^	36.1 ± 1.0^a^	34.0 ± 0.1^a^	0.36	0.14	0.31
	45	36.1 ± 1.5^a^	37.9 ± 1.0^ab^	36.6 ± 0.6^a^	37.9 ± 0.4^a^	0.84	0.21	0.84
	52	36.8 ± 1.2^a^	37.1 ± 1.2^ab^	36.6 ± 0.7^a^	36.1 ± 0.6^a^	0.67	0.92	0.77
	61	38.5 ± 0.6^a^	40.0 ± 0.5^a^	38.8 ± 0.8^a^	37.6 ± 1.1^a^	0.18	0.83	0.11
Mucosal fold height (µm)	15	43.6 ± 3.6^c^	42.5 ± 1.6^c^	42.1 ± 6.3^d^	50.1 ± 1.1^d^	-	-	-
	30	77.1 ± 2.1^b^	78.8 ± 1.0^b^	73.3 ± 2.3^c^	75.6 ± 3.7^c^	0.21	0.45	0.89
	45	92.6 ± 3.4^b^	87.2 ± 2.7^b^	90.6 ± 3.4^b^	96.5 ± 5.1^b^	0.36	0.94	0.15
	52	124.4 ± 7.3^a^	109.8 ± 5.2^a^	112.0 ± 3.7^a^	108.2 ± 6.7^ab^	0.33	0.21	0.44
	61	117.6 ± 3.7^a^	114.8 ± 6.1^a^	113.0 ± 3.3^a^	115.8 ± 1.3^a^	0.70	0.99	0.57

Values are given as mean ± SEM (*n* = 6). Within a row, *p*-values indicate the effect of PL level, BS supplement and their interaction (PL × BS) (two-way ANOVA). Within a column, different superscript letters indicate significant differences between age groups for a given parameter (Tukey’s HSD post hoc test, *p* < 0.05)

## Discussion

The liver is central to nutrient metabolism and energy homeostasis in fish, supporting lipid digestion through secretion of bile and storing energy reserves (Hoehne-Reitan and Kjørsvik 2004). Cod larvae hatch at an altricial stage with immature organs, and liver functionality during larval ontogeny remains poorly understood. Here, we examined liver development in Atlantic cod with emphasis on (1) biliary secretory capacity of phosphatidylcholine (PC) and bile salt (BS) mediated by the ATP-binding cassette (ABC) transporters Abcb4 and Abcb11, and (2) hepatic vacuolization as an indicator of maturation in energy storage. We further assessed how dietary phospholipid (PL) level and BS supplementation affect these processes and related histological biomarkers of nutritional status.

### Biliary ABC transporters

Both C219 and C494 produced positive immunoreactivity in cod, but only C219 labeled bile canaliculi, consistent with findings in rainbow trout (*Oncorhynchus mykiss*) (Curtis et al. 2000) and zebrafish (*Danio rerio*) (Machado et al. 2014). While C494 is specific for Abcb1, C219 cross-reacts with Abcb1, Abcb4 and Abcb11 (Georges et al. 1990; Childs et al. 1995). This suggests that Abcb4 and Abcb11, but not Abcb1, are expressed at the hepatocyte canalicular membrane. In agreement with this, phylogenetic analysis identified Abcb4 and Abcb11 in the cod genome, while Abcb1 has not been detected (Lukenbach et al. 2014). The same study found two cod Abcb11 paralogs, Abcb11a and Abcb11b. In zebrafish, *abcb11b* is liver-specific and shares greater amino acid sequence identity with human ABCB11 (70 %) than *abcb11a* (65 %), which is enriched in the intestine (Ellis et al. 2018). In contrast, zebrafish *abcb4* is expressed ubiquitously, including in the liver and intestine (Robey et al. 2021). It is therefore likely that the C219 signal in cod bile canaliculi reflects contributions from Abcb11b and Abcb4, while intestinal labeling may originate from Abcb11a and Abcb4. However, their relative contributions to the observed signals could not be determined in this study due to the lack of specific antibodies.

Bile canalicular labeling with C219 was evident already at 2 dph, indicating that biliary secretion via Abcb4 and/or Abcb11 is functional before exogenous feeding. Earlier studies in European seabass (*Dicentrarchus labrax*), gilthead seabream (*Sparus aurata*) and pikeperch (*Stizostedion lucioperca*) instead suggested exocytosis as the primary route of biliary secretion in larval fish (Mani-Ponset et al. 1994; Diaz et al. 1997). Our findings agree with reports of *abcb4* and *abcb11* expression in teleost embryos (Costa et al. 2012; Fischer et al. 2013) and with evidence that zebrafish *abcb11b* mutants fail to export BS from the liver and die prematurely (Ellis et al. 2018). The early role of Abcb11 in cod is further supported by the presence of bile salt-dependent lipase (BSDL) in the cod larval midgut shortly after hatching (Hoehne 1999), given that BSDL requires BS to function (Gjellesvik et al. 1992).

Canalicular C219 labeling increased by 68 % between 8 and 30 dph, suggesting enhanced capacity for bile secretion and possibly lipid digestion. A similar developmental increase in bile canaliculi has been reported in common carp (*Cyprinus carpio*) (Fishelson and Becker 2001). This aligns with previous findings in cod larvae, where intestinal lipid absorption increased at 17 dph (Kjørsvik et al. 1991) and specific BSDL content rose progressively from 2 to 31 dph (Hoehne 1999). Furthermore, trypsin production and activity increased markedly from 18 dph (Hjelmeland et al. 1984), consistent with the broader developmental pattern reported for pancreatic enzymes in teleost larvae (Hoehne-Reitan and Kjørsvik 2004). The plateau in C219 labeling beyond 30 dph suggests that the hepatobiliary system reaches functional maturity at this stage. These larvae averaged 9.2 mm SL and were approaching the start of metamorphic climax (Vo et al. 2016), when cod are capable of fully digesting and metabolizing lipids from formulated diets (Hamre et al. 2011). Therefore, canalicular C219 labeling appears to be indicative of hepatobiliary function as well as lipid digestive capacity in cod larvae.

Although Abcb11 is strongly implicated in bile secretion due to the importance of BS in lipid digestion, the increase in C219 labeling may also reflect higher Abcb4 abundance. If so, biliary PC delivery to the intestine could be limited during early development, potentially constraining intestinal lipoprotein assembly and increasing the requirements for dietary PC (Mansbach 1977; Li et al. 2015, 2016). However, this interpretation remains speculative, as C219 does not distinguish between Abcb4 and Abcb11. Furthermore, the substrate specificity of teleostean Abcb4 is still unresolved, with evidence pointing to functional homology to the mammalian multidrug transporter ABCB1 (Zaja et al. 2008; Fischer et al. 2013). Nonetheless, fish bile contains appreciable amounts of PC (Moschetta et al. 2005) that must be transported across the canalicular membrane, underscoring the need to clarify the role of Abcb4 in cod.

We also examined whether dietary PL level and BS supplementation would influence biliary ABC transporter abundance. The extent of dietary modulation of such transporters is not well studied in fish, although there has been a focus on effects of replacing fish meal with plant proteins (Kortner et al. 2013; Gu et al. 2014; Zhu et al. 2018; Yao et al. 2024). We found no significant effect on C219 labeling in bile canaliculi of cod, but larvae fed low-PL diets showed a non-significant 14 % higher signal than those fed high-PL diets. Because all diets were isolipidic, the higher proportion of neutral lipids in the low-PL diets may have induced Abcb11-mediated secretion of BS to enhance emulsification. This trend could also reflect higher biliary PC efflux via Abcb4 to support intestinal lipoprotein synthesis, in agreement with increased *abcb4* expression in mice fed high-lipid diets (Guo et al. 2015). It is therefore suggested that dietary lipid composition can potentially modulate the abundance of Abcb4 and/or Abcb11 in bile canaliculi of developing cod larvae.

Both antibodies produced cytoplasmic immunoreactivity in cod hepatocytes. For C219, this signal may represent intracellular trafficking of Abcb11. In WIF-B9 cells, a hybrid cell line derived from human hepatoma and rat fibroblasts that exhibits hepatocyte-like properties, ABCB11 localizes to tubulovesicular structures that migrate toward both membrane poles but fuse only at the canalicular surface (Wakabayashi et al. 2004). Such intracellular pools may help regulate Abcb11 abundance in response to BS secretion demands. In contrast, C494 showed diffuse cytoplasmic labeling across all tissue types, consistent with observations in Nile tilapia (*Oreochromis niloticus*) (Costa et al. 2013). This likely reflects cross-reactivity with pyruvate carboxylase (Rao et al. 1994), a mitochondrial enzyme involved in the Krebs cycle and gluconeogenesis (Valle 2017). The ubiquitous expression of pyruvate carboxylase in red seabream (*Pagrus major*) (Abe et al. 2004) aligns with the broad C494 labeling observed in our study.

### Hepatic vacuolization

Cod juveniles retain substantial amounts of hepatic lipids as TAG (Lie et al. 1986), whereas larvae store little lipid in their liver (Wold et al. 2009). The developmental timing of this shift has, however, not been described. In this study, hepatic vacuolization increased sigmoidally with larval size, from 2.3 % at ≤ 15 mm SL to 35.9 % at ≥ 23 mm SL. The sharpest rise occurred at 17.7 mm SL and was driven primarily by lipid rather than glycogen accumulation, indicating that hepatic lipid storage is initiated around the transition from climax to post-climax metamorphosis (15–20 mm SL) (Vo et al. 2016).

This transition does not appear to be linked to changes in digestive physiology, as canalicular C219 labeling peaked well before hepatic lipid accumulation, and the capacity to digest and metabolize lipids is already high at that stage (Hamre et al. 2011). Instead, the pattern aligns with changes in energy allocation strategy. Altricial fish larvae prioritize growth over storage (Di Pane et al. 2019), which results in a high mortality risk throughout the larval stage (Jordaan and Brown 2003). Such prioritization is consistent with the allometric growth of vital organs observed during early development (Osse et al. 1997; Sala et al. 2005; Wold et al. 2008; Gagnat et al. 2016). In our experiment, cod larvae grew allometrically in mass relative to length until 13.1 mm SL (Marthinsen et al. 2025), after which it became more isometric and matched that of juveniles and adults (Árnason et al. 2009). Simultaneously, the average specific growth rate declined from 8.9 to 5.9 % day^−1^, thereby allowing more energy to be stored. We therefore suggest that the timing of hepatic lipid accumulation in cod reflects a shift in energy allocation strategy around the transition from the larval to juvenile stage.

Ontogenetic shifts in energy metabolism may further explain the observed pattern. Vo et al. (2022) reported a marked upregulation of genes involved in glucose transport and glycolysis during stratified hyperplasia (7–10 mm SL), when muscle fiber proliferation is most pronounced (Vo et al. 2016). Active swimming during this period was suggested to enhance glucose metabolism in cod, while reliance on lipids to fuel muscle contraction appeared to decline thereafter. This shift occurred earlier in larvae fed copepods than in those fed rotifers, with copepod-fed larvae also showing faster growth and lower hepatic glycogen content (Karlsen et al. 2015; Kjørsvik, pers. comm.), indicating more efficient use of glucose as an energy substrate (Vo et al. 2022). Accordingly, the reduced PAS staining observed in highly vacuolated livers in our study might reflect improved glycogen utilization, thus enabling lipids to be diverted from β-oxidation toward storage.

Finally, we assessed whether diet influenced hepatic vacuolization. Previous studies have shown that dietary PLs may affect hepatic lipid content in fish larvae (Salhi et al. 1999; Caballero et al. 2004; Gisbert et al. 2005; Wold et al. 2009), and similar findings have also been reported for diets supplemented with BS (Jiang et al. 2018; Ruiz et al. 2023). In our study, vacuolization was slightly higher in the high-PL groups (22 %) compared to the low-PL groups (12 %) at 52 dph (17.9 mm SL), when the growth in hepatic lipid accumulation peaked. Although this difference was not statistically significant, this trend may indicate enhanced intestinal lipid transport to the liver via increased lipoprotein synthesis at higher dietary PL levels (Fontagné et al. 1998; Olsen et al. 2003). However, the presence of lipid droplets (1–10 µm) in enterocytes was unaffected by the dietary treatments and showed no apparent correlation with hepatic vacuolization. It is worth noting that the relatively small difference in PL levels may have limited our ability to detect diet-related effects.

### Nutritional status

Cod larval growth in this study was comparable to reports from pervious laboratory trials (Hansen et al. 2011; Karlsen et al. 2015; Øie et al. 2015), indicating adequate nutrition. To further evaluate nutritional status, we analyzed histological biomarkers in the liver and intestine (Gisbert et al. 2008). No significant dietary effects were observed, but hepatocyte nuclei decreased markedly between 15 and 30 dph. Since larger nuclei support higher metabolic activity through greater surface area (Ghadially 1997), this reduction suggests declining nutritional status, consistent with shrinking hepatocyte nuclei during starvation in larval pejerrey (*Odontesthes bonariensis*) (Strüssmann and Takashima 1990).

This decline coincided with the co-feeding period (17–34 dph), when *Artemia sp*. were gradually replaced by formulated diets (Marthinsen et al. 2025). Similarly, common whitefish (*Coregonus lavaretus*) larvae fed formulated diets had smaller hepatocyte nuclei than those fed zooplankton (Segner et al. 1988). Fish larvae generally prefer live prey (Fernández-Díaz et al. 1994), and their capacity to digest formulated diets is limited until the stomach becomes functional (Cahu and Infante 2001; Rønnestad et al. 2013). In cod, gastric glands required for pepsinogen and hydrochloric acid secretion appear around 39 dph (10 mm SL) (Kamisaka and Rønnestad 2011), while weaning in this study was completed at 35 dph. Therefore, the reduced nutritional status during co-feeding is likely a result of both decreased *Artemia sp*. abundance and an absence of gastric digestion. This effect was reversed by 52 dph, suggesting that larvae of ~ 18 mm SL were developed to effectively utilize the formulated diets.

From 52 to 61 dph, hepatocyte nucleus size declined again, with a significant reduction observed only in the LPL-BS group. This period corresponded with increased mortality toward the end of the trial, while hepatic vacuolization in the largest larvae (23–30 mm SL) plateaued at ~ 36 %, remaining well below the hepatic lipid levels reported for well-fed cod juveniles (Lie et al. 1986). This may reflect suboptimal feeding conditions and mobilization of hepatic lipid stores (Black and Love 1986), although necropsy revealed no clear cause of the mortality event. Overall, our results support earlier conclusions by Wold et al. (2009) that hepatocyte nucleus size is a sensitive indicator of nutritional status in cod larvae.

## Conclusion

This study provides the first immunohistochemical evidence of biliary ABC transporters in Atlantic cod, based on labeling with monoclonal antibodies C219 and C494. Both antibodies produced positive immunoreactivity in whole-larval tissue sections, but only C219 was expressed in bile canaliculi, consistent with Abcb4 and Abcb11, but not Abcb1. Canalicular labeling was detectable as early as 2 dph and increased between 8 and 30 dph, suggesting that biliary secretion is functional before the exotrophic stage and reaches maturity near the onset of metamorphic climax (9.2 mm SL). Hepatic vacuolization rose sigmoidally from 2.4 to 35.9 % with larval size, with the most rapid increase at the transition between climax and post-climax metamorphosis (17.7 mm SL). Histological staining confirmed that this increase was due to lipid and not glycogen accumulation, demonstrating that the liver begins to function as the primary site of lipid storage only as larvae approach the juvenile stage. Dietary treatments did not significantly affect canalicular C219 labeling, hepatic vacuolization, or larval nutritional status, suggesting that liver development in cod proceeds gradually independently of dietary PL level and BS supplementation under the tested conditions.

## Supplementary Information

Below is the link to the electronic supplementary material.

Supplementary file1 (DOCX 5720 KB)

## Data Availability

No datasets were generated or analysed during the current study.
