# Supplementary material for: Liver development in Atlantic cod (Gadus morhua L.) larvae: Histomorphological analysis of biliary ABC transporters and hepatic vacuolization
Source: Fish Physiol Biochem. 2025 Dec 22;52(1):4. doi: 10.1007/s10695-025-01623-7 (PMC12722359; doi:10.1007/s10695-025-01623-7)
Supplement: Supplementary file 1 — Supplementary file1 (DOCX 5720 KB) [file 10695_2025_1623_MOESM1_ESM.docx]

**Supplementary Information**


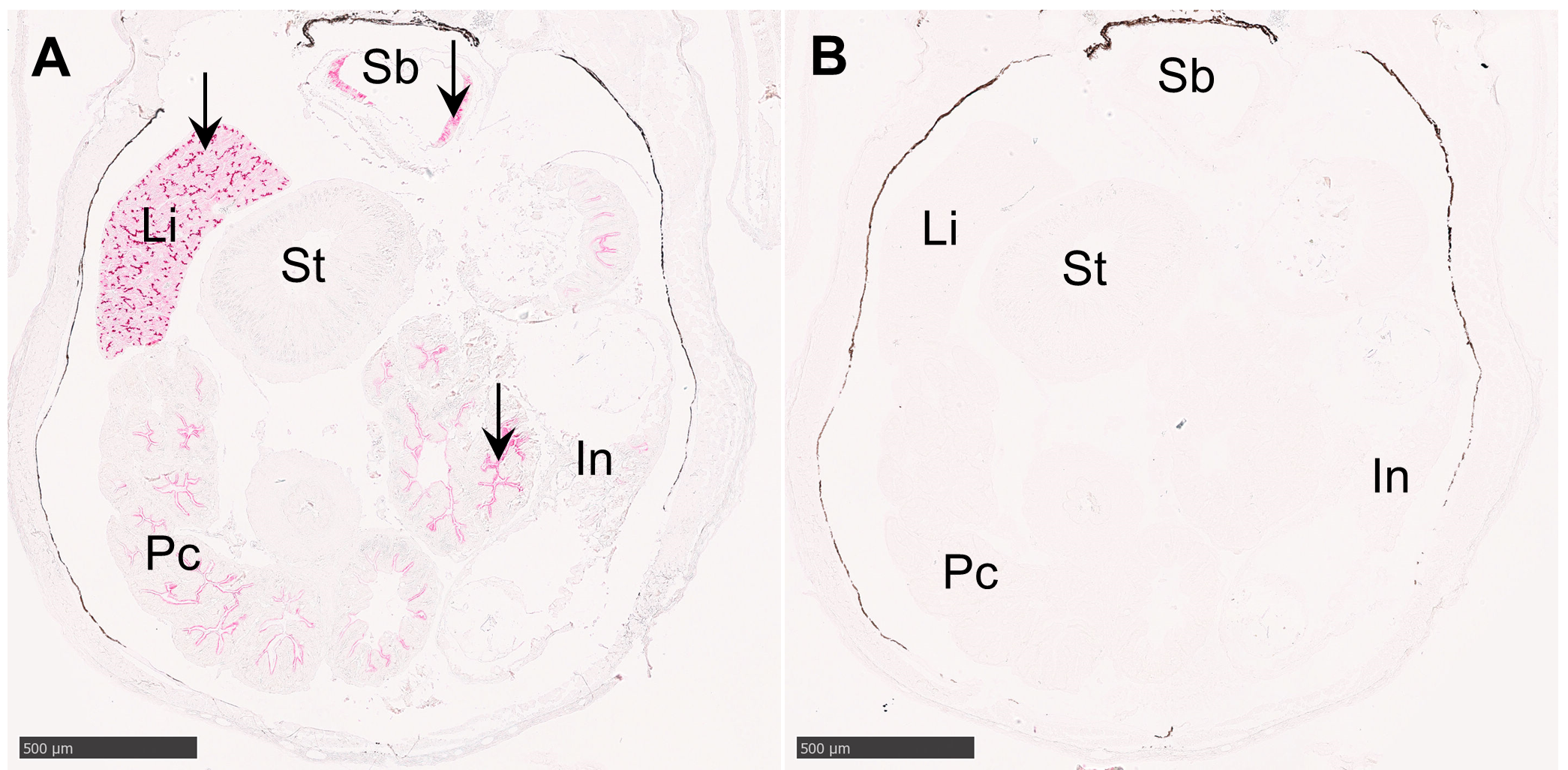


**Fig. S1** Transverse paraffin-embedded tissue sections of G. morhua at 60 dph. (A) Section labeled with C219. Positive labeling (arrows) is visualized with alkaline phosphatase and Vector Red. (B) Negative control without any positive labeling. In, intestine; Li, liver; Pc, pyloric caeca; Sb, swim bladder; St, stomach. Scale bars equal to 500 µm

**Table S1** C219 labeling of bile canaliculi in G. morhua liver sections (% area). The effect of normalization is expressed as the difference (L_n_ − L_m_) between measured labeling (L_m_) and normalized labeling (L_n_)

| Dph | Diet | Hepatic vacuolization (% area) | C219 labeling (% area) | | | *p*-value |
| --- | --- | --- | --- | --- | --- | --- |
|  |  |  | L*_m_* | L*_n_* | L*_n_* − L*_m_* |  |
| 8 | HPL | − | 6.0 ± 0.7 | − | − | − |
| 15 | HPL | 0.8 | 6.8 ± 0.6 | 6.8 ± 0.6 | 0.1 ± 0.0 | 0.013 |
| 30 | HPL | 1.8 | 9.9 ± 0.4 | 10.1 ± 0.4 | 0.2 ± 0.0 | < 0.001 |
| 45 | HPL | 2.1 | 7.8 ± 0.5 | 8.0 ± 0.4 | 0.2 ± 0.0 | < 0.001 |
| 60 | HPL | 37.4 | 5.6 ± 0.3 | 8.9 ± 0.5 | 3.3 ± 0.2 | < 0.001 |
| 60 | HPL-BS | 33.3 | 5.5 ± 0.3 | 8.2 ± 0.5 | 2.7 ± 0.2 | < 0.001 |
| 60 | LPL | 37.3 | 6.4 ± 0.5 | 10.2 ± 0.7 | 3.8 ± 0.3 | < 0.001 |
| 60 | LPL-BS | 35.4 | 5.9 ± 0.3 | 9.2 ± 0.5 | 3.3 ± 0.2 | < 0.001 |

Values are given as mean ± SEM (*n* = 6). Within a row, the *p*-value indicates if L_n_ > L_m_ (one-sided paired t-test)


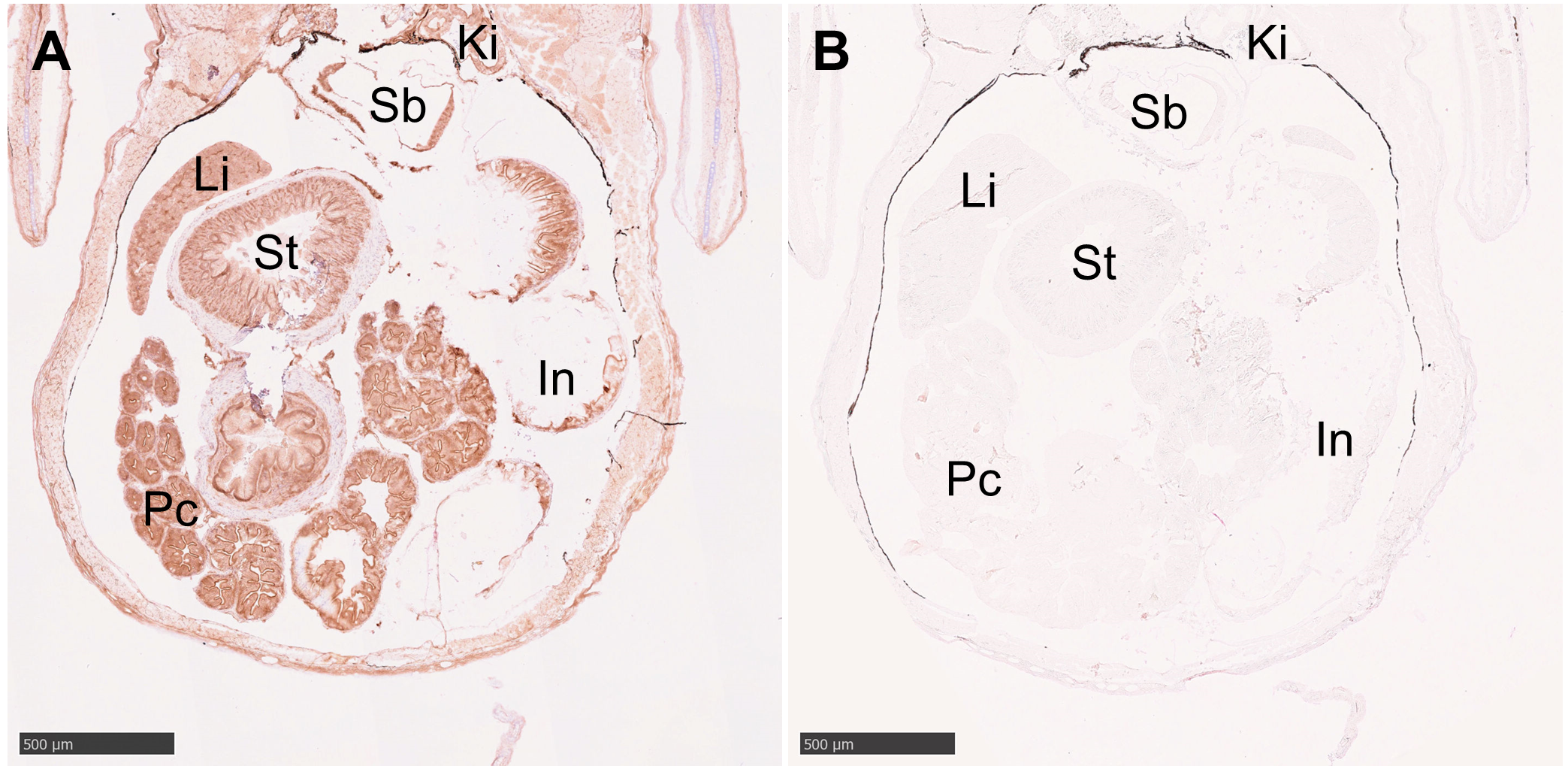


**Fig. S2** Transverse paraffin-embedded tissue sections of G. morhua at 60 dph. (A) Section labeled with C494. Positive labeling is visualized with horseradish peroxidase and DAB. (B) Negative control without any positive labeling. In, intestine; Ki, kidney; Li, liver; Pc, pyloric caeca; Sb, swim bladder; St, stomach. Scale bars equal to 500 µm
